# Surveillance of antimicrobial resistance by a global network of WHO collaborating centres

**DOI:** 10.2471/BLT.25.294384

**Published:** 2026-02-09

**Authors:** Anne Harant, Arina Zanuzdana, Sara Tomczyk, Tim Eckmanns, Insik Kong, Monica Lahra, Savannah Gill, Rene Hendriksen, Olga Perovic, Alejandra Corso, Sonja Löfmark Behrendtz, Lothar Kreienbrock, Majid Alshamrani, Stephan Harbarth, Marlieke de Kraker, Oskar Ekelund, Onur Karatuna, Carolien Ruesen, Silvia Bertagnolio, Yvan Hutin, on behalf of the WHO AMR Surveillance and Quality Assessment Collaborating Centres Network group

**Affiliations:** a Department of Infectious Disease Epidemiology, Robert Koch Institute, WHO Collaborating Centre for Antimicrobial Resistance, Consumption and Health Care-Associated Infections, Seestraße 10, Berlin, 13353, Germany.; b Antimicrobial Resistance Department, World Health Organization, Geneva, Switzerland.; c Department of Microbiology, New South Wales Health Pathology Randwick, Sydney, Australia.; d National Food Institute, Technical University of Denmark, Copenhagen, Denmark.; e Centre for Healthcare-Associated Infections, Antimicrobial Resistance and Mycoses, National Institute for Communicable Diseases, Johannesburg, South Africa.; f National and Regional Reference Laboratory for Antimicrobial Resistance, National Institute of Infectious Diseases, Buenos Aires, Argentina.; g Department of Communicable Disease Control and Preparedness, Public Health Agency of Sweden, Solna, Sweden.; h Institute for Biometry, Epidemiology and Information Processing, University of Veterinary Medicine, Hanover, Germany.; i Department of Infection Prevention and Control, Ministry of National Guard Health Affairs, Riyadh, Saudi Arabia.; j Infection Control Program, Geneva University Hospitals and Faculty of Medicine, Geneva, Switzerland.; k Department of Clinical Microbiology, Central Hospital, Växjö, Sweden.; l Centre for Epidemiology and Surveillance of Infectious Disease Control, National Institute for Public Health and the Environment, Bilthoven, Kingdom of the Netherlands.

## Abstract

Antimicrobial resistance remains one of the most pressing threats to health globally, with inappropriate antimicrobial use and inequitable access to quality-assured medicines compounding the challenge. In 2015, the World Health Organization (WHO) launched the Global Antimicrobial Resistance Surveillance System to strengthen global surveillance and provide data to inform responses. In support, in 2016 WHO established the AMR Surveillance and Quality Assessment Collaborating Centres Network, a global group of expert institutions that provide technical support and help WHO Member States build surveillance capacity. Between 2016 and 2025, the Network expanded from 18 to 36 institutions across 20 countries and its technical scope has grown to cover antimicrobial use surveillance, emerging antimicrobial resistance, fungal antimicrobial resistance and national action plans. This paper presents a process evaluation of the network, focusing on its development between 2016 and 2025, its contribution to tackling antimicrobial resistance and the challenges faced. Information from documentation and structured interviews indicate that the network played a central role in developing surveillance standards, strengthening laboratory and epidemiology capacity, and helping countries implement surveillance. Challenges persist: network representation is uneven across geographical regions, coordination of resources is limited, and the network’s impact is incompletely documented. To maximize its potential, the network must improve output tracking, expand membership from low- and middle-income countries and create subnetworks for specific topics and regions. The analysis offers lessons on how WHO collaborating centre networks can serve as strategic enablers of actions on global health, provided they are adequately supported and integrated into broader implementation frameworks.

## Résumé

La résistance aux antimicrobiens (RAM) reste l’une des menaces les plus préoccupantes pour la santé mondiale, et l’utilisation inappropriée des antimicrobiens ainsi que l’accès inégal à des médicaments de qualité garantie aggravent encore le problème. En 2015, l’Organisation mondiale de la Santé (OMS) a lancé le Système mondial de surveillance de la résistance aux antimicrobiens afin de renforcer la surveillance mondiale et de fournir des données pour éclairer les procédures de réaction à ce problème. À cet effet, l’OMS a créé en 2016 le Réseau de centres collaborateurs pour la surveillance et l’évaluation de la qualité de la RAM, un groupe mondial d’institutions expertes qui fournissent un soutien technique et aident les États membres de l’OMS à renforcer leurs capacités de surveillance. Entre 2016 et 2025, ce réseau est passé de 18 à 36 institutions dans 20 pays, et son champ d’action technique s’est élargi pour couvrir la surveillance de l’utilisation des antimicrobiens, la résistance émergente aux antimicrobiens, la résistance fongique aux antimicrobiens et les plans d’action nationaux. Cet article présente une évaluation du processus du réseau, en mettant l’accent sur son évolution entre 2016 et 2025, sa contribution à la lutte contre la résistance aux antimicrobiens et les défis rencontrés. Les informations issues de la documentation et des entretiens structurés indiquent que le réseau a joué un rôle central dans l’élaboration de normes de surveillance, le renforcement des capacités des laboratoires et de l’épidémiologie et l’aide apportée aux pays pour mettre en œuvre la surveillance. Des défis persistent toutefois: la représentation du réseau est inégale entre les régions géographiques, la coordination des ressources est limitée et l’impact du réseau n’est pas entièrement documenté. Pour maximiser son potentiel, le réseau doit améliorer le suivi des résultats, élargir sa composition aux pays à revenu faible et intermédiaire et créer des sous-réseaux pour des thèmes et régions spécifiques. L’analyse offre des enseignements sur la manière dont les réseaux de centres collaborateurs de l’OMS servent de catalyseurs stratégiques pour les actions en matière de santé mondiale, à condition qu’ils soient suffisamment soutenus et intégrés dans des cadres de mise en œuvre plus larges.

## Resumen

La resistencia a los antimicrobianos (RAM) sigue siendo una de las amenazas más apremiantes para la salud a nivel mundial. Además, el uso inadecuado de antimicrobianos y el acceso inequitativo a medicamentos con garantía de calidad agravan el desafío. En 2015, la Organización Mundial de la Salud (OMS) puso en marcha el Sistema Mundial de Vigilancia de la Resistencia a los Antimicrobianos para reforzar la vigilancia mundial y proporcionar datos que orienten las respuestas. Como apoyo, en 2016 la OMS estableció la Red de Centros Colaboradores para la Vigilancia de la RAM y la Evaluación de la Calidad, un grupo mundial de instituciones expertas que brindan apoyo técnico y ayudan a los Estados Miembros de la OMS a fortalecer la capacidad de vigilancia. Entre 2016 y 2025, la Red se amplió de 18 a 36 instituciones en 20 países, y su alcance técnico se extendió para abarcar la vigilancia del uso de antimicrobianos, la resistencia emergente a los antimicrobianos, la resistencia a los antimicrobianos en hongos y los planes de acción nacionales. En este artículo, se presenta una evaluación de procesos de la red, centrada en su desarrollo entre 2016 y 2025, su contribución para abordar la resistencia a los antimicrobianos y los desafíos enfrentados. La información procedente de la documentación y de entrevistas estructuradas indica que la red desempeñó un rol central en el desarrollo de normas de vigilancia, el fortalecimiento de la capacidad de laboratorio y epidemiología, y el apoyo a los países para implementar la vigilancia. Persisten desafíos: la representación de la red es desigual entre regiones geográficas, la coordinación de recursos es limitada y el impacto de la red está documentado de manera incompleta. Para maximizar su potencial, la red debe mejorar el seguimiento de productos, ampliar la membresía de países de ingresos bajos y medios y crear subredes para temas y regiones específicos. El análisis ofrece lecciones sobre cómo las redes de centros colaboradores de la OMS pueden actuar como habilitadores estratégicos de acciones en salud mundial, siempre que cuenten con el apoyo adecuado y se integren en marcos de implementación más amplios.

## ملخص

لا تزال مقاومة مضادات الميكروبات تُشكل أحد أخطر التهديدات الصحية على مستوى العالم، حيث أن الاستخدام غير المناسب لمضادات الميكروبات، وعدم المساواة في الحصول على الأدوية مضمونة الجودة، يؤديان لمضاعفة هذا التحدي. في عام 2015، أطلقت منظمة الصحة العالمية النظام العالمي لمراقبة مقاومة مضادات الميكروبات، بهدف تعزيز المراقبة العالمية وتوفير البيانات اللازمة لتوجيه الاستجابات. ودعمًا لذلك، أنشأت منظمة الصحة العالمية في عام 2016 شبكة المراكز المتعاونة لمراقبة مقاومة مضادات الميكروبات وتقييم الجودة، وهي مجموعة عالمية من المؤسسات ذات الخبرة التي تُقدم الدعم الفني وتُساعد الدول الأعضاء في منظمة الصحة العالمية على بناء قدراتها في مجال المراقبة. بين عامي 2016 و2025، توسعت الشبكة من 18 إلى 36 مؤسسة في 20 دولة، وتوسع نطاقها الفني ليشمل مراقبة استخدام مضادات الميكروبات، وظهور مقاومة جديدة لها، ومقاومة مضادات الميكروبات الفطرية لها، وخطط العمل الوطنية. تقدم هذه الورقة تقييمًا إجرائيًا للشبكة، مع التركيز على تطورها بين عامي 2016 و2025، ومساهمتها في التصدي لمقاومة مضادات الميكروبات، والتحديات التي واجهتها. تشير المعلومات من الوثائق والمقابلات الشخصية المنظمة إلى أن الشبكة لعبت دورًا محوريًا في وضع معايير المراقبة، وتعزيز القدرات المختبرية والوبائية، ومساعدة الدول على تطبيق المراقبة. ولا تزال هناك تحديات قائمة: فالتمثيل الشبكي غير متعادل بين المناطق الجغرافية، وتنسيق الموارد محدود، وتأثير الشبكة غير موثق بشكل كامل. ولتحقيق أقصى استفادة من إمكاناتها، يجب على الشبكة تحسين تتبع المخرجات، وتوسيع عضويتها لتشمل الدول ذات الدخل المنخفض والدخل المتوسط، وإنشاء شبكات فرعية لمواضيع ومناطق محددة. يقدم التحليل دروسًا حول كيفية مساهمة شبكات المراكز المتعاونة مع منظمة الصحة العالمية، في تمكين الإجراءات المتعلقة بالصحة العالمية بشكل استراتيجي، شريطة أن تحظى بالدعم الكافي وأن تتكامل في أطر أكثر وسعًا للتنفيذ.

## 摘要

抗微生物药物耐药性仍然是全球最紧迫的健康威胁之一，而抗微生物药物的不合理使用及有质量保证的药品的获取机会不均等进一步加大了此类挑战。世卫组织 (WHO) 于 2015 年启动了全球抗微生物药物耐药性监测系统，以加强全球监测并为制定应对措施提供数据支持。为了提供支持，通过汇集全球众多专家机构的力量，世卫组织于 2016 年设立了抗微生物药物耐药性 (AMR) 监测和质量评估合作中心网络，以提供技术支持和帮助世卫组织成员国建设监测能力。在 2016-2025 年期间，该网络从原先的 18 个机构扩展至遍及 20 个国家的 36 个机构，且其技术范畴也已扩大至涵盖抗微生物药物使用监测、新发抗微生物药物耐药性、真菌类抗微生物药物耐药性和国家行动计划。本文开展了针对该网络的过程评估，重点分析该网络在 2016-2025 年期间的发展情况、该网络在应对抗微生物药物耐药性问题方面的贡献及其所面临的各类挑战。从文件资料和结构化访谈中提取的信息表明，该网络在制定监测标准、提升实验室和流行病学能力并帮助各国落地监测工作方面发挥了核心作用。目前该网络仍面临诸多挑战：各区域的网络布局不均；资源协调能力有限；且网络的实际成效尚未形成完整记录。为了最大程度地发挥其潜能，该网络必须完善成果追踪机制、吸纳中低收入国家的机构以扩大成员规模并针对特定主题和区域设立分支网络。本次分析表明了世卫组织合作中心网络如何能够成为全球卫生行动的战略性赋能者——只要获得足够的支持并融入范围更广的实施框架之中。

## Резюме

Устойчивость к антимикробным препаратам является одной из наиболее серьезных угроз всемирному здравоохранению, при этом тяжесть проблемы усугубляется нерациональным использованием антимикробных препаратов и неравным доступом к качественным лекарственным средствам. В 2015 году Всемирная организация здравоохранения (ВОЗ) ввела в действие глобальную систему эпиднадзора за устойчивостью к антимикробным препаратам, чтобы укрепить глобальный эпиднадзор и обеспечить сбор данных для информированного реагирования. В качестве поддержки этой инициативы в 2016 году ВОЗ основала сеть сотрудничающих центров по эпиднадзору за устойчивостью к антимикробным препаратам (УПП) и оценке качества – международную группу специализированных учреждений, которые обеспечивают техническую поддержку и помогают государствам-членам ВОЗ наращивать потенциал надзорной структуры. В период с 2016 по 2025 год эта сеть расширилась с 18 до 36 учреждений в 20 странах. Ее технические возможности выросли и охватывают теперь надзор за использованием антимикробных препаратов, вновь возникающей устойчивостью к антимикробным препаратам и противогрибковым препаратам, а также отслеживание национальных планов действия. В данной статье представлена оценка процесса деятельности сети с особым вниманием к развитию в период между 2016 и 2025 годами, описан ее вклад в борьбу с устойчивостью к антимикробным препаратам и проблемы, с которыми пришлось столкнуться. Данные, полученные из документации и структурированных интервью, свидетельствуют о том, что сеть играла центральную роль в разработке стандартов надзора, укреплении лабораторного и эпидемиологического потенциала, а также в оказании помощи странам во внедрении систем надзора. Сохраняются, однако, и проблемы: представленность сети неравномерна по географическим регионам, координация ресурсов ограничена, а влияние сети задокументировано не в полной мере. Для максимального раскрытия своего потенциала сети необходимо улучшить отслеживание результатов деятельности, расширить участие стран с низким и средним уровнем дохода, а также создать подсети по отдельным тематическим направлениям и регионам. Анализ предлагает выводы о том, каким образом сети сотрудничающих центров ВОЗ могут выступать в роли стратегических факторов, способствующих действиям в области глобального здравоохранения, при условии их надлежащей поддержки и интеграции в более широкие рамки реализации.

## Introduction

In 2019, the World Health Organization (WHO) identified antimicrobial resistance as one of the top 10 public health threats globally and, subsequently, it was proposed that the risk of drug-resistant infections should be included in pandemic preparedness agreements.^1^^,^^2^ Addressing antimicrobial resistance requires a package of interventions in the health sector, in accordance with the One Health approach.^3^ The inappropriate use of antimicrobials is a major driver of antimicrobial resistance, while at the same time inadequate access to essential, quality-assured medicines remains a problem in many resource-constrained settings. Recognizing the urgent need for action, in 2024 many countries renewed and expanded their commitment to addressing antimicrobial resistance, first through a World Health Assembly resolution on antimicrobial resistance (WHA77.6)^4^ and, second, by endorsing a political declaration at a high-level meeting on antimicrobial resistance at the United Nations General Assembly, which was intended to guide the international response to antimicrobial resistance until 2030.^5^

As early as 2015, at the Sixty-eighth World Health Assembly, WHO Member States agreed a global action plan to tackle antimicrobial resistance (resolution WHA68.7),^6^ which included surveillance of, and monitoring the impact of interventions addressing, antimicrobial resistance. Several surveillance initiatives had already been established by then, such as: (i) the European Antimicrobial Resistance Surveillance Network in the European Union and European Economic Area; ^7^ (ii) the Central Asian and European Surveillance of Antimicrobial Resistance (CAESAR) network in WHO’s European Region;^8^ (iii) the Latin American and Caribbean Network for Antimicrobial Resistance Surveillance (ReLAVRA+) involving countries of the Pan American Health Organization;^9^ (iv) the WHO Advisory Group on Integrated Surveillance of Antimicrobial Resistance (AGISAR);^10^ and (v) the Global Foodborne Infections Network.^11^ However, investment has been variable or non-existent and approaches to the surveillance of antimicrobial resistance have differed.^12^

In 2015, WHO built on these initiatives by launching the Global Antimicrobial Resistance Surveillance System (GLASS), which provides a platform for, and a harmonized approach to, the collection, analysis, interpretation and reporting of data on antimicrobial resistance.^13^ In addition, WHO Member States recognized that the development and implementation of GLASS could be advanced by global collaboration and called for a network of collaborating centres. In response, WHO established the AMR Surveillance and Quality Assessment Collaborating Centres Network (hereafter referred to as the network) in 2016 to align surveillance activities, share expertise and promote consistent, high-quality data collection across regions.

In general, expert networks are vital for addressing complex global health threats, such as that posed by antimicrobial resistance, especially where the political will is limited and resources are constrained, as in low- and middle-income countries.^3^ In practice, WHO collaborating centres are public health or academic institutions recognized for their technical expertise. They play a key role in supporting the development and implementation of WHO’s programme goals and increasing the resources available for achieving those goals. The former WHO Director-General, Margaret Chan, underlined the importance of collaborating centres when she stated that everything WHO does relies on the expertise of hundreds of formal WHO collaborating centres and thousands of the best brains in science, medicine and public health.^14^ Since its launch, the network has made substantial contributions to the implementation of GLASS by supporting the development of technical standards and the publication of documents on best practice, by conducting quality assessments, and by supporting capacity-building for the collection of laboratory and epidemiological data. The network has also helped improve methods for collecting and managing data on antimicrobial resistance and antimicrobial use and for using these data to guide actions on antimicrobial resistance.

With recent international developments leading to decreased funding for WHO and for global health initiatives, the global community has increasingly recognized that WHO collaborating centres can provide a backbone of support for public health, both globally and in individual countries. Achieving antimicrobial resistance targets will require continuous innovation and collaboration, along with mechanisms for sharing the lessons learned. WHO’s AMR Surveillance and Quality Assessment Collaborating Centres Network is large and can provide a model for the establishment of other WHO collaborating centre networks.^15^^,^^16^

This paper presents the findings of a process evaluation of WHO’s AMR Surveillance and Quality Assessment Collaborating Centres Network that examined its development, its contribution to tackling antimicrobial resistance and the challenges faced. The evaluation was intended to help shape the network’s strategic direction and share insights to inform the design of future collaborating centre networks.

## Methods

Our process evaluation considered the activities and structure of WHO’s AMR Surveillance and Quality Assessment Collaborating Centres Network and its contribution to tackling antimicrobial resistance. Our analysis was structured using key elements of the SQUIRE 2.0 reporting framework,^17^ which emphasizes context, intervention descriptions, processes and outcomes. Our data sources included official documentation, such as workplans, designation and redesignation letters and annual reports, submitted by collaborating centres between 2016 and 2025.

Primary data collection involved structured, virtual meetings with each collaborating centre (Table 1) to map institutional expertise and ongoing antimicrobial resistance-related activities. In 2025, collaborating centre AUS-72 in Australia was supporting the coordination of the network and led the data compilation and analysis for this mapping exercise. In addition, data were extracted from the GLASS dashboard to assess progress in surveillance capacity,^18^ with country enrolment figures in GLASS for antimicrobial resistance and antimicrobial use serving as proxy indicators.

**Table 1:** Collaborating centres and contact personnel, WHO AMR Surveillance and Quality Assessment Collaborating Centres Network, 2024

WHO region, country and centre designation	Institution	Contact personnel
**African Region**		
South Africa, SOA-43	WHO Collaborating Centre for Antimicrobial Resistance, Centre for Healthcare-Associated Infections, Antimicrobial Resistance and Mycoses, National Institute for Communicable Diseases, Johannesburg	Nelesh Govender and Olga Perovic
**Region of the Americas**		
Argentina, ARG-30	WHO Collaborating Centre on the Rational Use of Medicines, University Center of Pharmacology, National University of La Plata, La Plata	Gustavo H Marin
Argentina, ARG-43	WHO Collaborating Centre on Antimicrobial Resistance Surveillance, National and Regional Reference Laboratory in Antimicrobial Resistance, National Institute of Infectious Diseases, Ministry of Health, Buenos Aires	Alejandra Corso, Cristina Canteros, Patricia Galarza and Carolina Carbonari
Costa Rica, COR-11	WHO Collaborating Centre for Antimicrobial Resistance Surveillance, *Centro Nacional de Referencia de Bacteriología, Instituto Costarricense de Investigación y Enseñanza en Nutrición y Salud*, Cartago	Grettel Chanto Chacón and María Antonieta Jiménez-Pearson
Mexico, MEX-33	WHO Collaborating Centre on Antimicrobial Resistance in Foodborne and Environmental Bacteria, General Directorate of Agrifood, Aquaculture and Fisheries Safety, National Service for Agrifood Health, Safety and Quality, Tecámac	Leandro David Soriano García, Mayrén Cristina Zamora Nava and Cindy Fabiola Hernandez Perez
United States, USA-304	WHO Collaborating Centre in Pharmaceutical Policy, Department of Global Health, Boston University School of Public Health, Boston	Veronika J Wirtz
United States, USA-417	WHO Collaborating Centre for Surveillance, Epidemiology and Control of Foodborne Diseases and Fungal Disease, National Center for Emerging Zoonotic and Infectious Diseases, Centers for Disease Control and Prevention, Atlanta	Tom Chiller and Alyson M Cavanaugh
United States, USA-449	WHO Collaborating Centre for Global One Health and Antimicrobial Resistance Initiatives, North Carolina State University Department of Population Health & Pathobiology, Raleigh	Paula J Fedorka-Cray, Megan Jacob and Siddhartha Thakur
United States, USA-451	WHO Collaborating Centre, Stanford University School of Medicine, Stanford	Marisa Holubar
United States, USA-458	WHO Collaborating Centre for International Monitoring of Bacterial Resistance to Antimicrobial Agents, National Center for Emerging Zoonotic and Infectious Diseases, Centers for Disease Control and Prevention, Atlanta	Dawn M Sievert and Jacob Clemente
United States, USA-484	WHO Collaborating Centre for Surveillance of Antimicrobial Resistance, Brigham and Women's Hospital, Boston	John Stelling and Ahmed Taha Aboushady
**South-East Asia Region**		
India, IND-161	WHO Collaborating Centre for Antimicrobial Resistance, One Health Trust, Bangalore	Ramanan Laxminarayan and Erta Kalanxhi
India, IND-99	WHO Collaborating Centre on Reference and Research on Fungi of Medical Importance, Department of Medical Microbiology, Postgraduate Institute of Medical Education and Research, Chandigarh	Arunaloke Chakrabarti and Shivaprakash M Rudramurthy
Thailand, THA-89	WHO Collaborating Centre for Antimicrobial Resistance Prevention and Containment, Clinical Epidemiology Unit, Department of Research, Faculty of Medicine Siriraj Hospital, Mahidol University, Bangkok	Visanu Thamlikitkul and Pinyo Rattanaumpawan
Thailand, THA-93	WHO Collaborating Centre for Antimicrobial Resistance Surveillance and Training, Department of Medical Sciences, General Bacteriology Section, National Institute of Health, Ministry of Public Health, Nonthaburi	Wacharaporn Kamjumphol
**European Region**		
Denmark, DEN-69	WHO Collaborating Centre for Antimicrobial Resistance in Foodborne Pathogens and Genomics, National Food Institute, Technical University of Denmark, Copenhagen	Rene Hendriksen, Susanne Karlsmose Pedersen and Jette Sejer Kjeldgaard
Germany, DEU-144^a^	WHO Collaborating Centre for Antimicrobial Resistance, Consumption and Health Care-Associated Infections, Department of Infectious Disease Epidemiology, Robert Koch Institute, Berlin	Tim Eckmanns, Sara Tomczyk, Anne Harant, Arina Zanuzdana and Muna Abu Sin
Germany, DEU-151	WHO Collaborating Centre for Research and Training for Health at the Human–Animal–Environment Interface, Institute for Biometry, Epidemiology and Information Processing, University of Veterinary Medicine, Hanover	Lothar Kreienbrock and Sandra Brogden
Netherlands, NET-42	WHO Collaborating Centre for Risk Assessment of Pathogens in Food and Water, Laboratory for Zoonoses and Environmental Microbiology, Centre for Infectious Disease Control, National Institute for Public Health and the Environment, Bilthoven	Ana Maria de Roda Husman
Netherlands, NET-71	WHO Collaborating Centre for Reference and Research on *Campylobacter* and Antimicrobial Resistance from a One Health Perspective, Department of Infectious Diseases and Immunology, Faculty of Veterinary Medicine, University of Utrecht, Utrecht	Jaap A Wagenaar
Netherlands, NET-89	WHO Collaborating Centre for Antimicrobial Resistance Epidemiology and Surveillance, Centre for Infectious Disease Control, Centre for Infectious Diseases Epidemiology and Surveillance, National Institute for Public Health and the Environment, Bilthoven	Susan van den Hof and Carolien Ruesen
Norway, NOR-11	WHO Collaborating Centre for Drug Statistics Methodology, Norwegian Institute of Public Health, Oslo	Irene Litleskare
Russian Federation, RUS-126	WHO Collaborating Centre for Capacity Building on Antimicrobial Resistance Surveillance and Research, Institute of Antimicrobial Chemotherapy of Smolensk State Medical University, Smolensk	Roman Kozlov and Mikhail Edelstein
Sweden, SWE-66^b^	WHO Collaborating Centre for Antimicrobial Resistance Containment, Department of Communicable Disease Control and Health Protection, Public Health Agency of Sweden, Solna	Sonja Löfmark Behrendtz
Sweden, SWE-72	WHO Collaborating Centre for Gonorrhoea and Other Sexually Transmitted Infections, National Reference Laboratory for Sexually Transmitted Infections, Department of Laboratory Medicine, Microbiology, Örebro University Hospital, Örebro	Magnus Unemo and Daniel Golparian
Sweden, SWE-74	WHO Collaborating Centre for Standardization of Antimicrobial Susceptibility Testing of Bacteria, Department of Clinical Microbiology, Central Hospital, Växjö	Oskar Ekelund, Onur Karatuna and Gunnar Kahlmeter
Switzerland, SWI-82	WHO Collaborating Centre on Infection Prevention and Control and Antimicrobial Resistance, Department of Internal Medicine, *Hôpitaux Universitaires de Genève*, Geneva	Stephan Harbarth and Marlieke de Kraker
United Kingdom, UNK-105	WHO Collaborating Centre for Reference & Research on Antimicrobial Resistance and Healthcare-Associated Infections, United Kingdom Health Security Agency, London	Katie Hopkins and Colin Brown
United Kingdom, UNK-323	WHO Collaborating Centre for Genomic Surveillance of Antimicrobial Resistance, Centre for Genomic Pathogen Surveillance, University of Oxford, Oxford	David Aanensen, Sophia David, Julio Diaz Caballero, Nabil-Fareed Alikhan, Diana Connor and Natacha Couto
**Eastern Mediterranean Region**		
Saudi Arabia, SAA-23	WHO Collaborating Centre for Infection Prevention and Control and Antimicrobial Resistance, King Abdulaziz Medical City, King Saud bin Abdulaziz University for Health Sciences, King Abdullah International Medical Research Center, Ministry of National Guard Health Affairs, Riyadh	Majid Alshamrani, Fayssal Farahat, Aiman El-Saed, Mohammed Alzunitan and Mohammed Abalkheel
**Western Pacific Region**		
Australia, AUS-150	WHO Collaborating Centre for Antimicrobial Resistance, Peter Doherty Institute for Infection and Immunity, University of Melbourne, Melbourne	Ben Howden, Courtney Lane and Chantel Lin
Australia, AUS-72^c^	WHO Collaborating Centre for Sexually Transmitted Infections and Antimicrobial Resistance, New South Wales Health Pathology Microbiology, Prince of Wales Hospital, Randwick	Monica M Lahra, Sebastiaan J van Hal, C Robert George and Savannah C Gill
China, CHN-120	WHO Collaborating Centre for Infectious Disease Epidemiology and Control, School of Public Health, University of Hong Kong Special Administrative Region, Hong Kong Special Administrative Region	Ben Cowling and Peng Wu
Japan, JPN-97	WHO Collaborating Centre for AMR Surveillance and Research, AMR Research Center, National Institute of Infectious Diseases, Tokyo	Motoyuki Sugai, Koji Yahara, Shizuo Kayama and Takuya Yamagishi
Japan, JPN-98	WHO Collaborating Centre for Prevention, Preparedness and Response to Antimicrobial Resistance, National Center for Global Health and Medicine, Tokyo	Norio Ohmagari, Nobuaki Matsunaga, Shinya Tsuzuki, Masahiro Ishikane, Yumiko Fujitomo, Shugo Sasaki and Ryuji Koizumi
Republic of Korea, KOR-110	WHO Collaborating Centre for AMR Reference and One Health Research, National Institute of Health, Korea Disease Control and Prevention Agency, Chungcheongbuk-do	Jung Sik Yoo, Gyung Tae Chung, Dong Chan Moon and Eun-Jeong Yoon

WHO: World Health Organization.

^a^ Network coordinator from 2019 to 2024.

^b^ Network coordinator from 2016 to 2019.

^c^ Network coordinator from 2024.

Network members provided structured feedback focusing on lessons learnt and on common challenges and opportunities. Additional insights were drawn from discussions during the fourth network meeting in Buenos Aires, Argentina, in 2023,^19^ from a dedicated session at the 2024 meeting of WHO’s Strategic and Technical Advisory Group for Antimicrobial Resistance,^20^ and from internal webinar series and annual meetings.

The analysis was guided by six questions related to the period from 2016 to spring 2025: (i) what were the objectives of the network; (ii) how was the network structured and governed and who were its members; (iii) what were the network’s main activities and areas in which technical contributions were made; (iv) what results and outputs were achieved; (v) what key challenges were encountered, both specific to the network and related to broader responses to antimicrobial resistance; and (vi) what opportunities exist to strengthen the network’s future contributions?

We analysed data from documents and interviews using a thematic approach guided by these six questions. Subsequently, six recurrent themes emerged during iterative analysis, which we consolidated as result categories: (i) network governance and financing; (ii) network membership; (iii) expanding areas of work; (iv) expert exchanges and networking; (v) network contributions; and (vi) addressing antimicrobial resistance challenges as a network. The last theme involved both systemic and network issues related to global antimicrobial resistance governance and literature.

## Results

In accordance with SQUIRE 2.0 reporting principles,^17^ we first provide a summary of the main contextual, structural and process characteristics of WHO’s AMR Surveillance and Quality Assessment Collaborating Centres Network. The network was established to strengthen the global coordination of antimicrobial resistance surveillance and quality assessment in response to requests from WHO Member States for support for GLASS. Subsequently, the network’s mandate has expanded to cover broader actions on antimicrobial resistance, including the implementation of national action plans and capacity-building across regions. The network links WHO and collaborating centres through technical activities, meetings and exchanges that promote standardization, shared learning, international collaboration and country engagement. 

### Network governance and financing

The network provides a mechanism connecting WHO and collaborating centres, with each organization having its own terms of reference. The Network Secretariat coordinates the network, and comprises WHO’s Antimicrobial Resistance Department and a specific collaborating centre designated on a rotational basis. The collaborating centres which fulfilled that role in the past were the Public Health Agency of Sweden (2016 to 2019), the Robert Koch Institute, Berlin, Germany (2019 to 2024) and New South Wales Health Pathology, Randwick, Australia (from 2024). New members of the network are identified and invited by WHO on the basis of their existing cooperation with the network and on their interest and expertise in priority areas of work. Workplans strategically aligned with WHO priorities are developed by agreement between WHO and all network members.

To date, the network has operated without dedicated funding. Activities have relied on in-kind contributions from collaborating centres, which have often been supported through national public health institutions or by project-based funding, including external quality assessments conducted by collaborating centre DEN-69 in Denmark for Fleming Fund projects such as EQuAsia and EQuAfrica.^21^ Notably, collaborating centre SOA-43, based at the National Institute for Communicable Diseases in South Africa, has contributed operational funding to WHO’s EQA African programme since 2002 and has participated in EQuAfrica as a consortium partner with the African Society for Laboratory Medicine.^22^

### Network membership

**Fig. 1:**
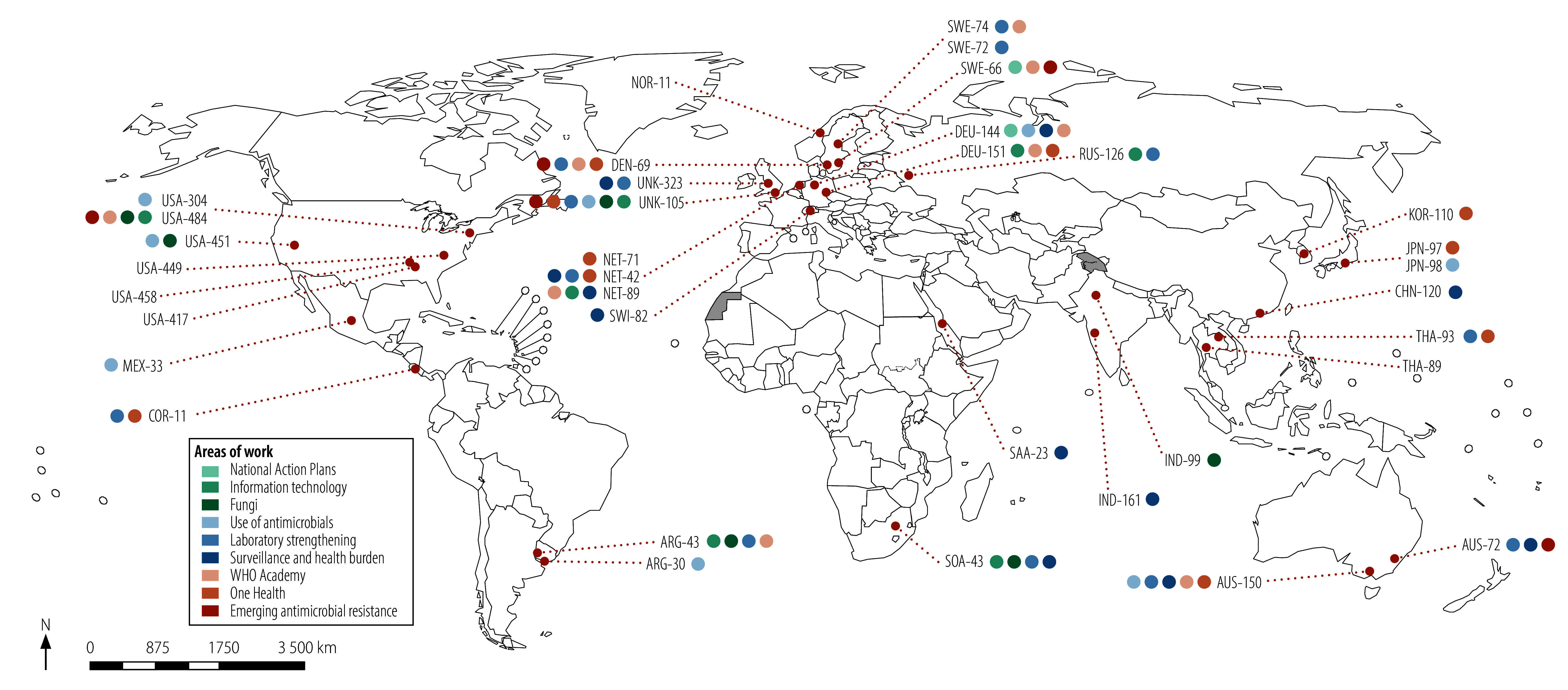
Collaborating centres and their areas of expertise, WHO AMR Surveillance and Quality Assessment Collaborating Centres Network, 2024

AMR: antimicrobial resistance; WHO: World Health Organization.

**Fig. 2:**
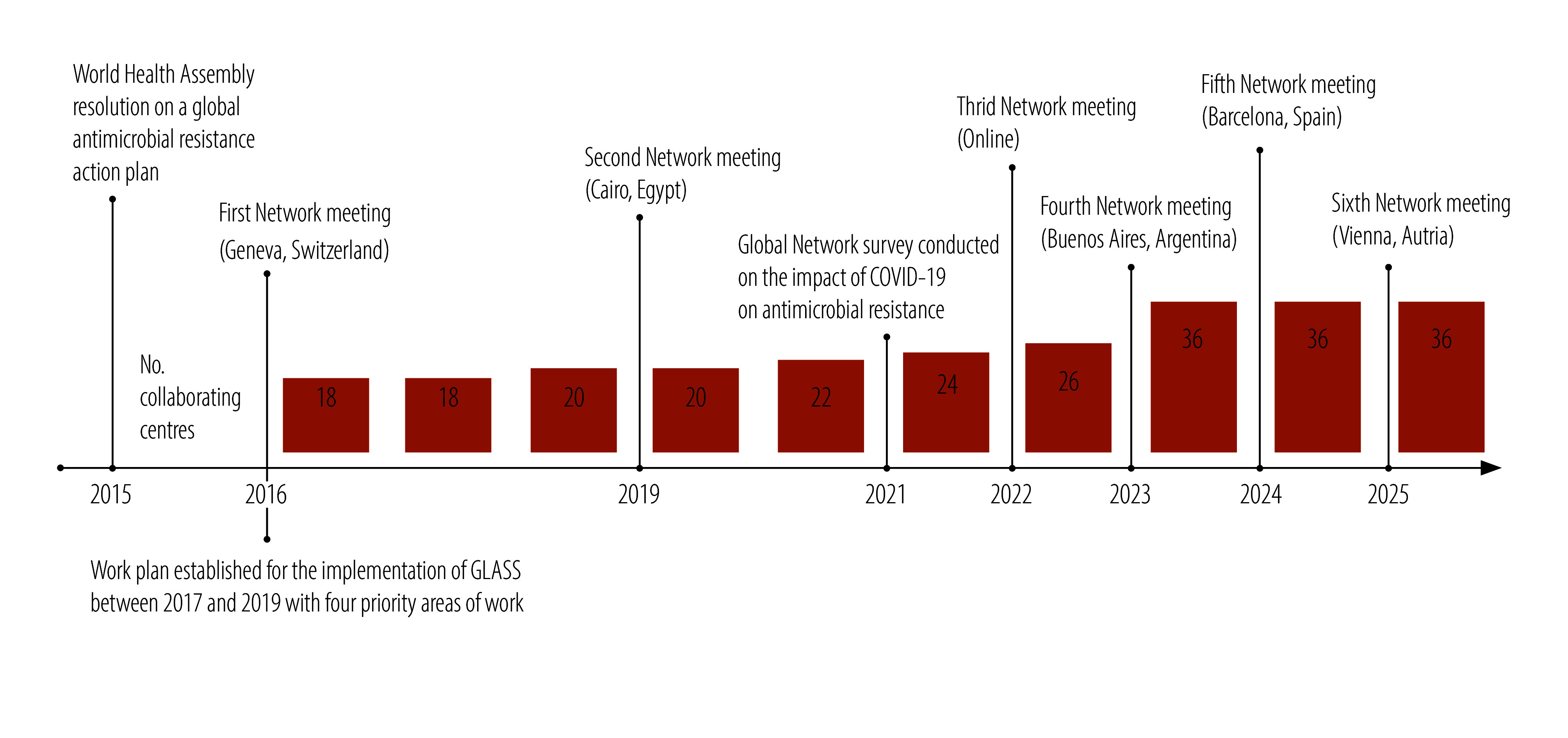
Timeline, evolution of the WHO AMR Surveillance and Quality Assessment Collaborating Centres Network, 2015–2025

AMR: antimicrobial resistance; COVID-19: coronavirus disease 2019; GLASS: Global Antimicrobial Resistance Surveillance System.

Notes: In 2015, the Sixty-eighth World Health Assembly adopted resolution WHA68.7, which approved a global action plan on antimicrobial resistance and requested the Director-General to establish the AMR Surveillance and Quality Assessment Collaborating Centres Network.^6^ A workplan was established for the implementation of the Global Antimicrobial Resistance Surveillance System (GLASS) between 2017 and 2019, which featured four priority areas of work: (1) surveillance; (ii) laboratories and microbiology; (iii) GLASS development and information technology; and (iv) understanding the impact of antimicrobial resistance. The fifth and sixth Network meetings took place as side events at European Society of Clinical Microbiology and Infectious Diseases global conferences. A global Network survey was conducted during the coronavirus disease 2019 pandemic to assess its impact on the surveillance, prevention and control of antimicrobial resistance.

In response to increasing calls for action against antimicrobial resistance from supporting WHO Member States, the network has grown in scope and size. Fig. 1 shows the geographical distribution of the 36 participating institutions from 20 countries and their technical expertise in 2024. Between 2016 and 2025, the network’s membership expanded from 18 collaborating centres with aligned activities to 36 (Fig. 2), thereby becoming a large technical network of WHO collaborating centres. Progressively, the new collaborating centres that joined the network provided expertise beyond surveillance in areas such as policy and stewardship. For example, one collaborating centre is dedicated exclusively to fungal antimicrobial resistance, whereas five others provide dual antimicrobial resistance expertise on both bacteria and fungi. Despite the proportion of collaborating centres located in low- and middle-income countries decreasing from 28% (5/18) in 2016 to 22% (10/36) in 2025, the overall number of centres increased. The regional distribution of collaborating centres is heterogeneous, ranging from 14 in the WHO European Region and 10 in the WHO Region of the Americas to one each in the WHO Eastern Mediterranean and African Regions.

In 2025, network mapping identified almost 200 experts qualified in science, medicine, public health, pharmacy and engineering from the six WHO regions. Their expertise covered antimicrobial resistance surveillance, laboratory strengthening, One Health, national action plan implementation, emerging antimicrobial resistance, information technology, antimicrobial use, mycology, bacteriology, infection prevention control, antimicrobial stewardship and external quality assurance.

### Expanding areas of work

**Fig. 3:**
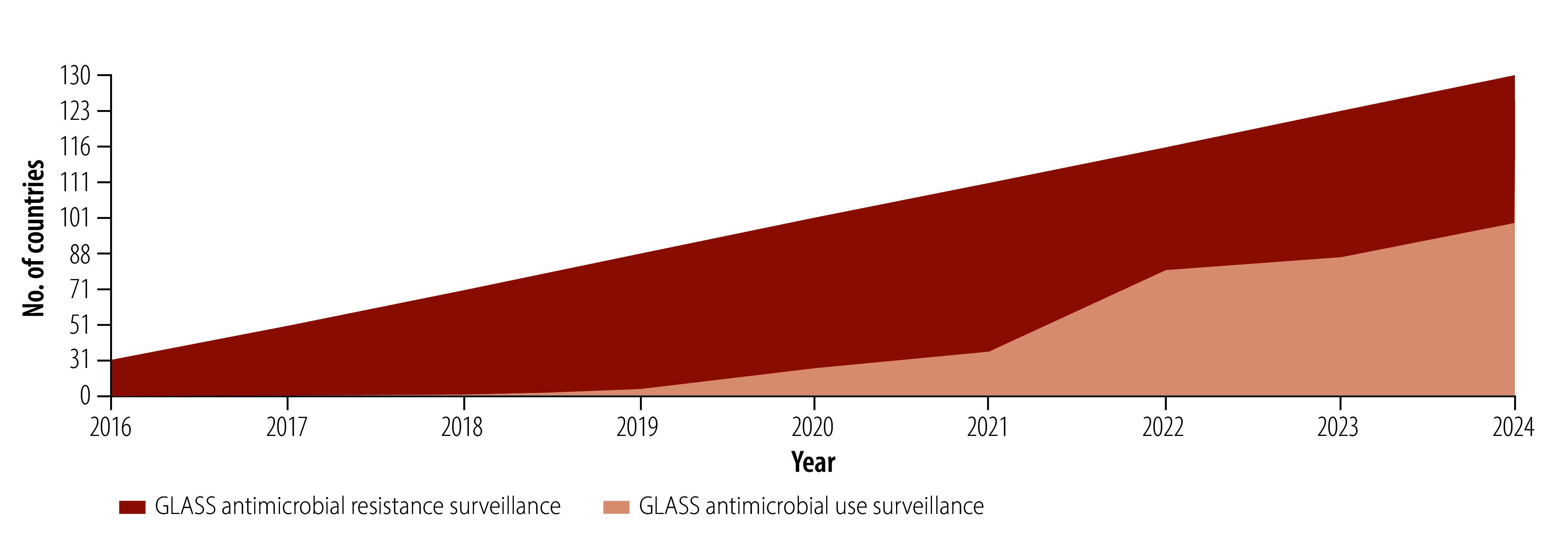
Timeline, country participation in the Global Antimicrobial Resistance Surveillance System, 2016–2024

GLASS: Global Antimicrobial Resistance Surveillance System; WHO: World Health Organization.

Notes: The figure shows the number of countries participating in the two core, routine, GLASS surveillance modules: (i) the antimicrobial resistance module; and (ii) the antimicrobial use module. Subsequently, GLASS technical modules were added over the years: (i) the GLASS emerging antimicrobial resistance reporting framework (GLASS-EAR);^23^ (ii) the GLASS early implementation protocol for the inclusion of *Candida* spp. (GLASS-FUNGI);^24^ (iii) the WHO method for point prevalence surveys on antibiotic use in hospitals (PPS-AMU);^25^ (iv) the GLASS method for estimating attributable mortality due to antimicrobial-resistant bloodstream infections (BURDEN);^26^ and (v) WHO integrated global surveillance of extended-spectrum β-lactamase-producing *Escherichia coli* species using a One Health approach.^27^

Under GLASS, different surveillance activities are grouped into technical modules. The two core, routine, data surveillance modules are: (i) the antimicrobial resistance module, which was launched in 2015; and (ii) the antimicrobial use module, which was launched in 2020. Between 2016 and 2024, the number of countries, territories and areas enrolled in GLASS antimicrobial resistance surveillance grew from 31 to 130 and the number enrolled in GLASS antimicrobial use surveillance grew from zero to 98 (Fig. 3). Moreover, GLASS has expanded both geographically and in scope, with increases in the number of technical modules and the number of standardized methods used for surveillance. This expansion has promoted a shift from surveillance approaches based solely on laboratory data towards one that seeks to include epidemiological, clinical and population data and that involves antimicrobial use data and data from One Health platforms. Nonetheless, the proportion of countries enrolled in GLASS that submit actionable surveillance data on antimicrobial resistance or use varied across regions. Several network members contributed to One Health initiatives such as the Tricycle project but their overall focus remained on strengthening human health surveillance.

In 2025, network collaborating centres were involved in nine technical areas of work and in three overarching areas of work (Table 2). Their activities were led and supported by different individual collaborating centres and were coordinated by WHO technical officers responsible for particular areas of work.

**Table 2:** Areas of work, WHO AMR Surveillance and Quality Assessment Collaborating Centres Network, 2024

Area of work	No. collaborating centres involved (*n* = 36)
**Technical area of work**	
Developing, implementing and monitoring national action plans on antimicrobial resistance for WHO Member States in accordance with a people-centred approach^28^	5
Diagnostics for bacterial and fungal infections and laboratory strengthening (i.e. WHO Antimicrobial Resistance Diagnostic Initiative)^29^	11
Strategies and methods for antimicrobial resistance surveillance and health burden estimation	8
GLASS information technology platform and data management tools for antimicrobial resistance and use surveillance	5
Reporting framework for emerging antimicrobial resistance^23^	6
Antimicrobial use surveillance^30^	9
Surveillance and diagnosis of fungal infections, including antifungal-resistant infections	6
Surveillance of antimicrobial resistance in the context of the One Health approach (i.e. the human–animal–environment interface)^31^	8
Training and capacity-building through the WHO Academy^32^	11
**Overarching area of work**	
Network engagement	2
Mapping activities and expertise	2
Country support^a^	28

AMR: antimicrobial resistance; GLASS: Global Antimicrobial Resistance Surveillance System; WHO: World Health Organization.

^a^ More than two thirds of collaborating centres were involved in country support.

Note: Most activities of the WHO AMR Surveillance and Quality Assessment Collaborating Centres Network focused on surveillance, the health burden of antimicrobial resistance and laboratory strengthening.

### Expert exchanges and networking

Regular meetings were held between network members and WHO’s Antimicrobial Resistance Department to provide opportunities for the exchange of information on activities and priorities and to generate synergies. Since 2016, the format of network annual meetings has transformed from single large events to hybrid meetings or side events attached to large conferences, with the aim of reducing travel requirements and carbon footprints. During the coronavirus disease 2019 (COVID-19) pandemic, collaborating centres conducted a global survey among countries enrolled in GLASS to assess the global effects of COVID-19 on antimicrobial resistance. Two hybrid meetings were held successfully in 2024 and 2025 during European Society of Clinical Microbiology and Infectious Diseases global conferences, which are habitually attended by many network members. Collaboration and engagement have also been fostered through online platforms. For example, a series of internal web seminars has taken place on different topics, during which collaborating centres were able to discuss their experiences, best practice, challenges and solutions to challenges.

### Network contributions

The network contributed to the development and expert review of GLASS manuals.^13^^,^^26^^,^^33^ In addition, network experts provided critical guidance for GLASS standards and methods and were involved in a range of efforts to improve data quality and build laboratory and epidemiology capacity,^34^^,^^35^ including: (i) developing an online GLASS platform for data submission; (ii) providing guidance for national reference laboratories on national antimicrobial resistance surveillance;^36^ (iii) developing methods for detecting and reporting colistin resistance;^37^ (iv) developing molecular methods for antimicrobial resistance diagnostics to enhance GLASS;^38^ (v) developing whole-genome sequencing for antimicrobial resistance surveillance;^39^^,^^40^ (vi) developing GLASS modules, including those on the surveillance of antimicrobial resistance in *Candida* spp. and *Neisseria gonorrhoeae*; and (vii) developing GLASS’s emerging antimicrobial resistance reporting framework.^23^^,^^24^ They also responded to WHO’s request to support the implementation of GLASS across countries, for example, by conducting field assessments of national surveillance capacities, training laboratory staff and providing technical advice.

Network contributions to GLASS methods for the surveillance of national antimicrobial use included developing: (i) WHO’s method for point prevalence surveys on antibiotic use in hospitals;^25^ (ii) the GLASS guide for national surveillance systems on monitoring antimicrobial consumption in hospitals; and (iii) training on GLASS’s methods for national surveillance of antimicrobial consumption. In addition, experts from collaborating centres also: (i) served as trainers for GLASS and other antimicrobial resistance surveillance workshops worldwide, with the aim of increasing countries’ laboratory and epidemiology capacity for the surveillance of antimicrobial resistance and use;^10^ (ii) provided expertise on antimicrobial resistance external quality assessment for all WHO regions; and (iii) contributed to the people-centred approach to addressing antimicrobial resistance in human health.^28^

### Addressing challenges as a network

Box 1 summarizes the key antimicrobial resistance challenges that could benefit from a coordinated network approach, and Table 3 provides more details on the context of these challenges and on strategies for addressing them. Despite the progress achieved through GLASS, persistent challenges remain, particularly for low- and middle-income countries where inequities in universal health coverage (UHC) limit access to diagnosis and treatment. Moreover, the implementation of quality-assured systems for microbiology laboratories has frequently been hindered by gaps in infrastructure, a shortage of skilled personnel and consumables, and the high cost of diagnostic materials, which can exceed the cost of antimicrobial treatment, thereby undermining both diagnostic stewardship and antimicrobial resistance surveillance. Together, these factors, combined with high staff turnover and the low perceived value of diagnostic testing among clinical and managerial staff, have contributed to the underuse of microbiological testing and laboratory-based surveillance.

Box 1 **Antimicrobial resistance challenges and addressing these through a network**

***Making antimicrobial resistance a priority for global health***

(i) Promote the integration of antimicrobial resistance into global health and pandemic preparedness frameworks; (ii) support unified action through commitments by the World Health Assembly and United Nations General Assembly; and (iii) foster cross-sectoral partnerships to sustain political attention and coordinated responses.

***Securing sustainable financing and support for implementing national action plans on antimicrobial resistance***

(i) Advocate funding for combatting antimicrobial resistance at global and national levels by leveraging collaborating centres’ links with policy-makers; (ii) promote the integration of funding for antimicrobial resistance into national budgets; and (iii) share evidence on, and success stories about, tackling antimicrobial resistance to strengthen countries’ commitment and sustain investment.

***Bridging gaps in access to affordable diagnostics and integrating antimicrobial resistance into health system strengthening and UHC initiatives***

(i) Promote global and regional mechanisms to improve access to, and the affordability of, antimicrobial resistance diagnostics; (ii) support the local production of, and capacity-building for, antimicrobial resistance diagnostics; and (iii) align antimicrobial resistance activities with broader health system strengthening and UHC initiatives.

***Strengthening diagnostic, laboratory and epidemiological capacity on antimicrobial resistance worldwide***

(i) Promote the adoption of GLASS standards and digital tools; (ii) expand training and mentorship in laboratory and data management; (iii) improve the integration of clinical and surveillance data; and (iv) support quality assurance and sector-specific antimicrobial resistance surveillance capacity-building.

***Providing coherent messages about the antimicrobial resistance surveillance approaches best suited to local contexts, with the aims of improving patient management and informing policy decisions***

(i) Facilitate inclusive discussions on tailoring surveillance methods to local capacities; (ii) engage clinicians and professional societies on linking antimicrobial resistance data with patient care; (iii) promote the use of local data on antimicrobial resistance for setting targets; and (iv) provide expert feedback to WHO on optimizing surveillance strategies.

***Improving the quality and coverage of antimicrobial use surveillance***

(i) Advocate for antimicrobial use surveillance as a core component of antimicrobial resistance monitoring; (ii) promote practical One Health strategies for surveillance systems; and (iii) strengthen the surveillance capacity of low- and middle-income countries through training, the use of digital data systems, and stakeholder collaboration on sustainable financing and policy support.

***Improve collaboration within, and the representativeness of, the Antimicrobial Resistance Surveillance and Quality Assessment Collaborating Centres Network***

(i) Enhance collaboration and communication through the use of diverse formats and regional engagement; (ii) foster the active participation of network members and promote inclusiveness; (iii) build capacity in low- and middle-income countries; (iv) evaluate network effectiveness; and (v) strengthen partnerships to align efforts and support the implementation of national action plans.

GLASS: Global Antimicrobial Resistance Surveillance System; UHC: universal health coverage; WHO: World Health Organization.

**Table 3:** Commentary on antimicrobial resistance challenges and addressing these through a network

Challenge	Context and barriers to improvement	Addressing the challenge
Making antimicrobial resistance a priority for global health	Despite the considerable health and economic burden of antimicrobial resistance globally, political commitment and sustainable funding for antimicrobial resistance activities remain limited in many settings.^41^ This is compounded by: (i) the complexity and interdisciplinary nature of antimicrobial resistance; (ii) low public, political and professional awareness; (iii) inconsistent regulations across countries; and (iv) a lack of intersectoral coordination and financial support. Within the One Health framework, each health sector may have distinct priorities and antimicrobial resistance may not be consistently a primary focus for all involved parties	(i) Advocate the inclusion of antimicrobial resistance as a global health threat in pandemic preparedness instruments and agreements, and advocate funding for antimicrobial resistance surveillance and interventions; (ii) present concrete strategies for the prevention and control of antimicrobial resistance and cooperate with high-level stakeholder groups to unify efforts following the adoption of World Health Assembly resolution WHA77.6 and the United Nations General Assembly high-level meeting on antimicrobial resistance;^4^^,^^5^ and (iii) mobilize partnerships, and facilitate dialogue, between different health sectors (including One Health stakeholders in public health, clinical medicine and laboratories) in countries with active network members
Securing sustainable financing and support for implementing national action plans on antimicrobial resistance	In 2024, a total of 170 countries had a national action plan on antimicrobial resistance but only about 10% of those plans were funded.^42^ Although national strategies existed for specific disease control programmes, for example, for HIV, tuberculosis or malaria, there was often little or no historical precedent for organizational structures with a dedicated budget that focused on coordinated antimicrobial resistance activities	(i) Apply concerted advocacy for antimicrobial resistance at global, regional and national levels; (ii) utilize the strong connections between collaborating centres and national policy-makers to raise awareness of the importance of antimicrobial resistance along with other urgent public health concerns; (iii) lobby for the inclusion of funds for antimicrobial resistance in national budgets and agendas to improve effective implementation of national action plans; (iv) exchange information on successful strategies and design use-cases to show the value of surveillance and interventions with the aim of increasing country participation; and (v) increase the body of local data on antimicrobial resistance to reinforce the importance of, and the financial case for, tackling antimicrobial resistance
Bridging gaps in access to affordable diagnostics and integrating antimicrobial resistance into health system strengthening and UHC initiatives	In many low-resource settings, there is a low level of diagnostic stewardship and poor access to supplies for diagnostics and surveillance, including material for pathogen culture and antimicrobial susceptibility testing. For example, procurement shortages and supply chain interruptions can affect the availability of blood culture bottles, culture media and antibiotic susceptibility test discs. Antimicrobial resistance activities are often seen as isolated and disease-specific, without a clear link to UHC and health system strengthening. From the patient's perspective, out-of-pocket expenses related to diagnostics can be prohibitively high and patients may have a lack of awareness or agency that prevents them actively requesting diagnostic tests from clinicians	(i) Advocate for global and regional procurement mechanisms, supply chains and financing for antimicrobial resistance diagnostics and for supportive policy; (ii) support the local production of laboratory consumables; (iii) work with global and national stakeholders on shared goals to integrate antimicrobial resistance responses into broader health system strengthening and UHC initiatives; and (iv) provide training in microbiology laboratory processes, such as antimicrobial susceptibility testing and molecular and whole-genome sequencing diagnostics for antimicrobial resistance
Strengthening diagnostic, laboratory and epidemiological capacity on antimicrobial resistance worldwide	The low utilization of bacteriological diagnostics in many low-resource settings may not only be due to cost and supply chain issues, but may also stem from the absence of well-established diagnostic stewardship capacity characterized by a lack of: (i) clinician awareness; (ii) high-quality pre-analytic and sampling procedures; (iii) quality-assured laboratory testing; (iv) timely post-analytical procedures for managing results and feedback; and (v) trust between clinicians and laboratory staff. In many countries, external quality assessment has still not been established, which has led to low-quality antimicrobial resistance data being collected nationally and submitted to GLASS. Efforts to improve diagnostic testing and to train staff are complicated by the complexity of multiple sampling, pathogen diversity and the range of pathogen–drug resistance combinations involved as well as by the quantity and quality of related epidemiological information. Lessons learned from other infectious diseases, such as HIV infection, tuberculosis and malaria, could be helpful, where relevant. However, substantial efforts are also needed to develop specific capacities and approaches for antimicrobial resistance, given its complexity^43^	(i) Promote the application of GLASS methodologies and other standardized guidance to ensure consistency across facilities and national and regional antimicrobial resistance surveillance activities; (ii) support GLASS-related training to coordinate efforts and exchange knowledge on capacity development in countries with collaborating centres; (iii) support countries in strengthening their laboratory information systems and apply information technology to improve the data used locally and submitted to GLASS; (iv) support countries in working towards integrating epidemiological and patient-based data, including sample and isolate results, to guide patient management and infection prevention and control;^44^ (v) promote the exchange of expertise and ongoing mentorship to further develop capacity in diagnostic stewardship, phenotypic and genotypic testing of antimicrobial resistance, and data analytical methods; (vi) provide access to external quality assurance programmes to collaborating centre partner countries and all interested WHO Member States; and (vii) strengthen One Health surveillance, including integrated surveillance across human, animal and environmental sectors
Providing coherent messages about the antimicrobial resistance surveillance approaches best suited to local contexts, with the aims of improving patient management and informing policy decisions	There is increasing interest in a range of approaches to antimicrobial resistance surveillance, from traditional culture-based antimicrobial resistance diagnostics to genomic surveillance and wastewater surveillance. However, these different approaches could be implemented in conflict with each other in a competitive and uncoordinated way instead of in a collaborative way that would improve patient management.^44^^,^^45^ Surveillance data are also important for informing policy, for setting high-level targets and for supporting political declarations	(i) Foster open discussions, stakeholder consultations and guideline development on which approaches are best suited to the local context, goals and resources; (ii) actively engage with clinicians and incorporate their perspectives – surveillance should generate evidence that can be used for patient management and for developing treatment guidelines, both of which necessitate outreach to clinical and microbiological societies; (iii) advocate the use of surveillance data for developing and informing high-level targets set, for example, at United Nations General Assemblies;^43^ (iv) develop ways of using local data, including those not reported to GLASS; and (v) continue providing expert feedback to WHO on surveillance methods globally and locally, including the surveillance of resistance to novel antibiotics^45^
Improving the quality and coverage of antimicrobial use surveillance	Many countries still lack the tools and knowledge required to establish and maintain basic surveillance systems for monitoring antimicrobial use. In many low- and middle-income countries, it is difficult to obtain good-quality, comprehensive data from all relevant health sectors. Antimicrobial use surveillance should be prioritized in national action plans	(i) Advocate antimicrobial use surveillance as an essential tool for understanding antimicrobial resistance and problems with access to antimicrobials, excess antimicrobial use and global shortages; (ii) present strategies for setting up antimicrobial use surveillance systems in the context of the One Health approach; (iii) cooperate with key stakeholders in advocating for global and regional procurement mechanisms, supply chains and financing for antimicrobial use surveillance and for supportive policy; and (iv) support capacity-building in low- and middle-income countries to improve competence in antimicrobial use surveillance, including establishing electronic health record systems and refining data collection and reporting processes
Improve collaboration within, and the representativeness of, the AMR Surveillance and Quality Assessment Collaborating Centres Network	The network includes institutions from different geographical regions with a diverse range of expertise, with network members contributing their time, effort and expertise on a voluntary basis. Initiatives have been undertaken to enhance communication and to encourage active participation in the planning and implementation of workplans. However, continuous, active effort is needed. The representation of collaborating centres from low- and middle-income countries is currently insufficient and should be improved	(i) Work with WHO to explore different means of network collaboration and communication; (ii) find new opportunities for face-to-face meetings, for example, in side events at global conferences; (iii) convene working groups at global or regional levels; (iv) increase the activity and engagement of network members through greater involvement in agenda development and network decision-making; (v) provide and facilitate educational training in low- and middle-income countries for interested network members; (vi) evaluate the network’s effectiveness using standardized tools; (vii) explore inclusion mechanisms for network members to ensure network activity and priorities reflect diverse geographical regions and country income levels; (viii) explore partnerships with agencies whose activities are aligned with network goals to minimize duplication and expedite results; (ix) strengthen subnetworks within WHO regions to help network Member States implement national action plans on antimicrobial resistance; and (x) improve partnerships with local groups of governmental institutions and offices of international organizations to develop context-specific solutions for antimicrobial resistance data collection, analysis and reporting

AMR: antimicrobial resistance; GLASS: Global Antimicrobial Resistance Surveillance System; HIV: human immunodeficiency virus; UHC: universal health coverage; WHO: World Health Organization.

## Discussion

Our analysis found that WHO’s AMR Surveillance and Quality Assessment Collaborating Centres Network contributed meaningfully to advancing global antimicrobial resistance surveillance, particularly through supporting GLASS. The network’s high level of technical expertise, interdisciplinary composition, global reach and trusted relationships with WHO and national stakeholders provided strong foundations for developing and promoting WHO norms and standards, for supporting countries’ implementation of antimicrobial resistance surveillance and national action plans, and for influencing policy discussions. In particular, the relationships built between collaborating centres and WHO enabled collaborations with countries to be effective and sustainable. Collaborating centres also had strong connections to key stakeholders in antimicrobial resistance, such as policy-makers, public health authorities and health funders, in both countries hosting collaborating centres and partner countries. Together, national and international partners contributed to a global network that has advocated, and will continue to advocate, for antimicrobial resistance surveillance and foster the financing and implementation of surveillance both regionally and globally.

Despite the progress made, there remain substantial challenges that will require coordinated, long-term efforts. For example, antimicrobial use surveillance faces several challenges, such as difficulty obtaining comprehensive data from all health sectors, particularly because of the existence of informal and unregulated markets for medicines and a reliance on paper-based records. Poor data quality and inadequate coverage of antimicrobial use make it difficult to draw inferences. Moreover, surveillance competence is limited in many countries and public health officials may not recognize the value of submitting data on antimicrobial use to GLASS, which will result in underreporting and gaps in global understanding of antimicrobial use patterns. In synergy with WHO, network collaborating centres can play a central role in increasing policy-makers’ awareness of the importance of data on antimicrobial use, in enhancing surveillance competencies and in refining data collection processes.

Other challenges for antimicrobial resistance surveillance are associated with broad responses to antimicrobial resistance and UHC, including access to antimicrobials and diagnostics. Through its connections with policy-makers, the network can play an important role in increasing funding for, and the implementation of, national action plans on antimicrobial resistance. This work may involve finding practical ways of, and sustainable financing for, improving the quality of data on antimicrobial resistance and use at local, regional and global levels. However, capacity-building and diagnostic training can be time-consuming and their benefits can dissipate if support is not provided after project completion. Sustaining the skills and competencies acquired requires long-term investment in communication, training and global partnerships. The alignment of in-kind contributions from network members and sustainable national support and funding would enable network activities to function synergistically rather than in parallel, thereby improving their global impact.

In 2024, a landmark report from the African Union on antimicrobial resistance explained how constrained resources affect responses.^46^ Whereas the primary factor driving antimicrobial resistance in high-income countries is antimicrobial use, African countries are affected by a lack of access to clean and safe water, poor water, sanitation and hygiene programmes, and a struggle to implement adequate infection prevention measures and biosecurity. Addressing these problems requires strong political commitment, cross-sectoral actions and more predictable funding. The network is well-positioned to support the implementation of WHO’s strategic priorities in this context: collaborating centres could take advantage of their national connections to help translate global frameworks (for example, a people-centred approach) into national action plans and to bridge persistent gaps in responses to antimicrobial resistance.

To address the challenge of antimicrobial resistance more broadly, WHO should facilitate multilateral collaboration within the network to address countries’ complex needs in an agile way that is strategically aligned with WHO’s priorities. Strong relationships between network members and low- and middle-income countries can enable regular cooperation despite the uneven global distribution of collaborating centres. However, the unbalanced regional distribution of collaborating centres also needs to be addressed.

Regular in-person meetings representing all network members are complicated by geographical and financial considerations and the associated carbon footprint. To address this, in-person meetings could be linked to conferences or other events, and smaller, topic-specific subnetwork meetings, for example on fungal antimicrobial resistance, could enable more focused technical cooperation. These logistical challenges mirror broader global issues in the governance of antimicrobial resistance responses, where sustained interactions and trusted partnerships are essential for coordinated, effective national responses.^41^ Improving interactions, and building trustworthy relationships, between WHO and network members can have a cumulative impact on reducing the burden of antimicrobial resistance in individual countries.

Our analysis has one main limitation. We observed a temporal association between an increase in the number of network collaborating centres and an increase in countries participating in GLASS, but were unable to determine precisely how the work of the collaborating centres contributed to greater country participation. Although the network was set up to track outputs, this was challenging in practice. From a result-based management perspective, we did not sufficiently document process steps, which limited our ability to demonstrate the impact of collaborating centre engagement.

In summary, the network of collaborating centres expanded between 2016 and 2025 and surveillance of antimicrobial resistance and use was strengthened globally, which demonstrates that a coordinated network can make a substantial contribution to achieving WHO’s priorities. The growth and activity of the network were associated with both gains and limitations: progress has been supported, particularly in surveillance, but challenges remain for the coordination, scope and sustainability of the Network.

Key lessons for similar networks include: (i) ensuring the geographical representation of the network and the themes covered are sufficient to align technical expertise with each country’s needs; (ii) monitoring the network’s output so that measurable results can be demonstrated; and (iii) creating topic-specific and regional subgroups to improve the coordination of support for individual countries.

Recent geopolitical changes and the contraction in overseas development assistance may lead to some collaborating centres experiencing a reduction in funding and, consequently, in their ability to engage in network activities. So far, however, the network has continued to expand, with members showing a strong commitment and a readiness to assume greater responsibility for advancing the antimicrobial resistance agenda and maintaining key surveillance functions. Although each network will develop its own dynamic, we hope our findings will inspire the development of more WHO collaborating centre networks, which could become powerful platforms for meeting complex global health goals through trusted partnerships, strategic alignment and sustained investment.
